# The Diagnosis and Treatment of Branch Retinal Vein Occlusions: An Update

**DOI:** 10.3390/biomedicines13010105

**Published:** 2025-01-05

**Authors:** Diana-Maria Darabuş, Rodica Georgiana Dărăbuş, Mihnea Munteanu

**Affiliations:** 1 Department of Ophthalmology, “Victor Babeş” University of Medicine and Pharmacy, 300041 Timișoara, Romania; diana.darabus@umft.ro (D.-M.D.); mihnea.munteanu@umft.ro (M.M.); 2 Doctoral School, “Victor Babeş” University of Medicine and Pharmacy, 300041 Timișoara, Romania

**Keywords:** branch retinal vein occlusion, vascular endothelial growth factor, macular edema, anti-VEGF therapy, gene therapy, peptide-based agents, small-molecule inhibitors

## Abstract

Branch retinal vein occlusion (BRVO) is a common retinal vascular condition and a significant contributor to vision loss worldwide, particularly in middle-aged and elderly populations. This review synthesizes current knowledge on the epidemiology, pathogenesis, and clinical features of BRVO, alongside recent advancements in diagnostic and therapeutic strategies. BRVO is approximately four times more prevalent than central retinal vein occlusion (CRVO) and often leads to significant vision impairment. By focusing on BRVO, this review aims to address the specific challenges and advancements in its diagnosis and management. The pathophysiology of BRVO is complex, involving factors such as venous compression, inflammation, and increased levels of vascular endothelial growth factor (VEGF). Diagnostic approaches such as optical coherence tomography (OCT) and fluorescein angiography are highlighted for their roles in assessing disease severity and guiding treatment decisions. Therapeutic interventions, including laser photocoagulation, anti-VEGF therapy, and intravitreal corticosteroids, are critically evaluated, emphasizing emerging treatments such as gene therapy, peptide-based agents, and small-molecule inhibitors. Despite advancements in management strategies, the recurrence of macular edema and treatment resistance remain significant challenges. Continued research is essential to refine therapeutic protocols and improve long-term visual outcomes for patients with BRVO.

## 1. Introduction

Retinal diseases significantly contribute to visual impairment and blindness globally, particularly among middle-aged and elderly individuals, though they can affect people of all ages [1,2,3]. Retinal vein occlusion (RVO) is the second most prevalent vascular retinal disease, following diabetic retinopathy [4,5,6], and it ranks as the fifth leading cause of blindness [7,8,9]. Moreover, RVO is a frequent cause of sudden, unilateral, painless vision loss [8,10,11,12]. RVO was first described by Liebreich in 1855 and later by Michel in 1878 [13]. Subsequently, branch retinal vein occlusion (BRVO) was described by Leber in 1877 and Oeller in 1896 [14]. RVO is a vascular condition marked by venous dilation and tortuosity, accompanied by secondary intraretinal hemorrhage, cotton wool spots, ischemia, optic disc and macular edema, diffuse retinal edema [15], neovascularization, and neovascular glaucoma [16,17,18]. RVO arises from partial or complete obstruction due to thrombosis in the central, hemi-central, or branched retinal veins [19]. The clinical manifestations of RVO are predominantly influenced by the levels of vascular endothelial growth factor (VEGF) in the vitreous and retina, which result from retinal ischemia [20].

RVO encompasses a spectrum of vascular disorders, with BRVO and CRVO as its primary subtypes. While CRVO involves the central retinal vein, BRVO occurs at arteriovenous crossings, often due to arterial compression. BRVO is significantly more common, accounting for approximately 4.42 cases per 1000 individuals compared to 0.8 cases for CRVO [5]. The distinct epidemiological and clinical characteristics of BRVO justify its focused exploration.

Previous reviews have detailed the natural history and management of BRVO, emphasizing its prevalence and distinct challenges compared to CRVO. The current review builds on this foundation, incorporating recent advancements in diagnostics and therapeutics to provide a comprehensive update [9].

This review employs a systematic approach to ensure a systematic and transparent methodology. A literature search was conducted across databases including PubMed, Scopus, and Web of Science. Inclusion criteria encompassed studies focusing on BRVO diagnosis, pathogenesis, and treatment published in peer-reviewed journals between 2000 and 2024. Exclusion criteria included studies without clear differentiation between BRVO and CRVO, non-peer-reviewed articles, and studies with sample sizes below 10 patients.

## 2. Epidemiology

RVO is a common vascular retinal disorder, with BRVO being more prevalent than CRVO. Global prevalence rates vary by region and ethnicity. The precise epidemiological data on RVO is unclear [21,22]; however, studies conducted in various populations indicate that the prevalence of RVO ranges from 5.2 to 16 per 1000 individuals [14].

For instance, studies among Caucasians aged 40 years and older report a BRVO prevalence ranging from 0.6% to 1.1% [21,23]. In Asian populations, prevalence is at approximately 0.76 per 100 individuals, and BRVO is six times more common than CRVO [4]. The Bhaktapur Retina Study in Nepal identified an RVO prevalence of 2.95%, with BRVO comprising 2.74% of cases [4,24]. In a 2016 study conducted in Korea, the prevalence of RVO was reported at 0.7%, with 0.6% for BRVO, noting no gender differences [25]. Pooled data from the International Consortium for Eye Diseases indicate an overall RVO prevalence of 5.20 per 1000 individuals, with BRVO affecting 4.42 per 1000, suggesting a global burden of approximately 16 million cases. These findings suggest that approximately 16 million people worldwide suffer from this vascular disorder, with BRVO being about four times more common than CRVO [5,10]. Ethnic and regional variations highlight the influence of genetic and environmental factors, with prevalence rates being higher in Asian populations compared to Western countries. These findings underscore the importance of early risk stratification and management in high-risk populations.

BRVO is commonly classified based on its anatomical location, such as temporal or nasal, with temporal BRVO being significantly more prevalent and clinically impactful. Temporal BRVO often affects the macula due to its proximity, resulting in substantial visual impairment, while nasal BRVO is frequently asymptomatic and may go undiagnosed. This asymptomatic nature introduces potential bias in prevalence studies, leading to an underreporting of cases [5,14]. Comprehensive screening methods are essential to accurately identify and report both symptomatic and asymptomatic cases, thus providing a more accurate representation of the disease burden.

Commonly associated factors with retinal venous occlusion include advanced age, hypercholesterolemia, hypertension, and a history of heart attacks [26]. Other epidemiological studies also highlight diabetes mellitus, congestive heart failure, and cerebrovascular disease as risk factors for RVO [24,27,28]. Cardiovascular risk factors are particularly significant as the primary pathogenic mechanism of BRVO involves arterial stiffness leading to venous compression within the common adventitial sheath [10,29].

## 3. Pathogenesis and Evolution

The pathological mechanisms underlying RVO remain not fully understood. The pathogenesis of RVO appears to be multifactorial [10]. Two primary consequences of RVO that contribute to diminished visual acuity (VA) are macular edema (ME) and retinal ischemia [20].

BRVO commonly occurs at arteriovenous intersections, where it is believed that the atherosclerotic artery exerts mechanical compression on the vein. Despite this compression, the venous lumen often remains preserved [30,31]. Additionally, local constriction of the vein may result from alterations in the biochemical environment [7]. The anatomical positioning of crossing vessels was assessed in 106 eyes with BRVO and it was discovered that in 99% of the affected eyes, the artery was situated anterior to the vein at the site of the blockage [32]. Building on these findings, it was suggested that mechanical obstruction of the vein by the rigid artery at arteriovenous (AV) crossings can induce turbulent blood flow, which in turn may damage the vascular endothelium and intima [10,33]. Such mechanisms underscore the significance of anatomical and structural evaluations in understanding the etiology and progression of BRVO.

It has been observed that hyperviscosity, resulting from elevated hematocrit levels, is associated with BRVO [34]. Additionally, dysregulation of the thrombosis–fibrinolysis balance is another hematological condition implicated in the pathogenesis of BRVO. However, the precise role of coagulation factors in the development of RVO remains ambiguous [10,35].

RVO initiates both inflammatory and hypoxic responses, which contribute to the disruption of the blood–retinal barrier [36] and lead to the development of ME. Prolonged ME results in apoptosis of photoreceptor cells in the macular region, ultimately causing irreversible loss of visual acuity in patients with BRVO [37,38,39].

The progression of BRVO is influenced by factors such as the location and extent of the occlusion, the integrity of arterial perfusion in the affected area, and the effectiveness of collateral circulation development [10,40]. Research has identified two distinct clinical forms of BRVO: major BRVO and macular BRVO. Studies examining the natural history of visual outcomes for both types in untreated eyes have shown that VA and visual fields generally improve to varying extents without intervention in most cases. However, eyes with macular BRVO experienced less improvement in VA compared to those with major BRVO. The mean duration for resolution of macular edema was 21 months for major BRVO and 18 months for macular BRVO [1].

Although many eyes with BRVO show improvements in VA without treatment, some eyes do not experience any improvement. The lack of improvement may be attributed to factors similar to those observed in ischemic CRVO: ischemic damage to macular retinal ganglion cells, pigmentary degeneration, and the formation of an epiretinal membrane resulting from prolonged macular edema [41,42,43,44,45].

Eyes with BRVO and substantial capillary non-perfusion may develop retinal neovascularization and vitreous hemorrhage; however, they are significantly less likely to develop neovascular glaucoma compared to eyes with CRVO or hemi-central retinal vein occlusion (hemi-CRVO). Over time, the acute process typically resolves, allowing for the clearance of hemorrhages and exudates. Collateral vessels may form between the superior and inferior retinal veins, facilitating recovery of visual acuity through improved venous drainage and the resolution of retinal edema and ischemia. The prognosis for vision loss due to BRVO is influenced by the extent of non-perfusion and the location of the occlusion [46,47].

The Branch Vein Occlusion Study (BVOS) group identified the severity of the occlusion and the extent of ischemia as crucial prognostic factors for the final visual acuity outcome in BRVO [33,46]. It has been confirmed that, in general, visual acuity improved or remained stable in approximately 80% of eyes with BRVO [48]. Additionally, it has been noted that while visual acuity in BRVO can improve without intervention, significant improvement beyond 20/40 is less frequently observed [7,9].

In conclusion, the pathogenesis of BRVO is driven by a combination of mechanical compression, vascular turbulence, hypoxia, and inflammation, leading to macular edema and retinal ischemia as primary causes of visual impairment. The progression and outcomes of BRVO depend on the extent of venous occlusion, arterial perfusion, and the development of collateral circulation. While some cases resolve spontaneously, persistent complications like neovascularization and chronic macular edema emphasize the importance of early diagnosis and tailored therapeutic approaches.

## 4. Diagnosis

Some authors recommend that a comprehensive approach be adopted for patients with RVO, which includes a detailed medical history, a thorough eye examination, and retinal imaging. Due to the associated systemic risk factors, involvement of an internist in the management of RVO patients is advised [49]. Following the ocular examination and retinal imaging, it is essential to differentiate between BRVO and CRVO, assess macular edema, estimate the extent of retinal ischemia, and evaluate for retinal and/or iris neovascularization. The initial history should address the location and duration of vision loss, current medications, and relevant medical and ocular history. The initial examination should include assessments of visual acuity, pupillary responses, identification of fine abnormal vessels in the iris using slit-lamp biomicroscopy, intraocular pressure (IOP), gonioscopy before dilation, funduscopic binocular evaluation, and examination of the peripheral retina and vitreous [46].

### 4.1. Clinical Diagnosis

The diagnosis of RVO is based on both subjective and objective signs presented by the patients. Subjectively, patients may report a variable decrease in visual acuity accompanied by a painless eye. Although vision loss can be severe, the onset is typically subacute, in contrast to the sudden vision loss characteristic of central retinal artery occlusion. In cases of severe venous stasis, infarction may occur due to the slowed retinal blood flow, often resulting in a dramatic clinical appearance referred to as the “blood and thunder” fundus. Objective examination of the fundus reveals venous vascular dilatations, as well as both superficial (flame-shaped) and deep (spot-shaped) hemorrhages, alongside retinal edema associated with vascular stasis. Hemorrhages are predominantly intraretinal but may also be preretinal or subretinal. If only a specific retinal sector is affected, it may indicate a venous branch occlusion, with observable changes occurring upstream of the crossing point [14,50,51].

BRVO can be diagnosed in various locations, including the nasal or temporal, superior, or inferior quadrants. Nasal BRVO, often discovered incidentally, typically does not affect visual acuity due to its distance from the macula. In contrast, temporal BRVO often impacts the macula lutea and is characterized by a decrease in visual acuity. BRVO located in the superotemporal quadrant is prone to spreading to the macula due to the gravitational effect on intraretinal fluid. Some temporal BRVOs may be asymptomatic. A hallmark of BRVO is venous dilation peripheral to the site of occlusion, which commonly occurs at the arteriovenous junction. Another characteristic feature of BRVO is retinal hemorrhage. Hemorrhages in BRVO are predominantly located in the area drained by the occluded branch. Vision loss is generally secondary to ME or ischemia. Additionally, retinal edema is present in the affected areas, and if retinal ischemia is involved, cotton-wool spots may be observed [14,52].

### 4.2. Paraclinical Diagnosis

Imaging tests can complement the clinical examination and enhance patient management. Various ocular imaging modalities have been explored and developed to assess RVO, retinal capillary nonperfusion, and retinal neovascularization (RNV).

Traditional eye examination and fluorescein angiography remain valuable for visualizing retinal vascular changes and assessing areas of nonperfusion. However, these approaches have been complemented by more advanced technologies in recent years.

Optical coherence tomography (OCT) has become a cornerstone for diagnosing and managing BRVO. Its high-resolution, cross-sectional imaging capability allows for precise evaluation of retinal layers, enabling clinicians to assess macular edema and structural damage with accuracy. Beyond its role in initial diagnosis, OCT is a critical instrument for monitoring treatment response and tracking disease progression over time [51]. OCT offers high-resolution imaging that allows clinicians to evaluate key structural biomarkers associated with visual prognosis in BRVO. Changes observable on OCT include central macular thickness, a vital indicator of macular edema severity and a reliable predictor of visual outcomes. A reduction in central macular thickness is often correlated with significant visual acuity improvement, underscoring its importance in monitoring therapeutic efficacy. Additionally, hyper-reflective foci, identifiable as discrete reflective spots within the retinal layers, serve as markers of retinal inflammation and oxidative stress, which are associated with advanced disease stages and poorer visual prognoses. Foveal intraretinal hemorrhage, another OCT-detectable feature, reflects vascular damage within the macula and contributes to visual impairment and delayed recovery. OCT also enables the assessment of the ellipsoid zone’s integrity, a critical marker of photoreceptor health. Disruption of this zone is linked to compromised visual recovery, whereas its restoration is strongly associated with improved outcomes. The structural integrity of the inner retinal layers, also evaluable via OCT, provides further prognostic information, as damage to these layers often results in sustained visual deficits. Lastly, vitreomacular adhesion, visible as tractional changes at the macula, exacerbates macular edema and may impede recovery unless resolved. These biomarkers, detectable through OCT, offer invaluable insights into disease mechanisms and therapeutic responses, highlighting the pivotal role of OCT in the comprehensive evaluation and management of BRVO [53].

Optical coherence tomography angiography (OCTA) represents a major breakthrough in retinal imaging. Unlike fluorescein angiography, OCTA is non-invasive and capable of providing detailed visualization of retinal and choroidal microvasculature. This technique enables clinicians to differentiate ischemic and non-ischemic BRVO by identifying capillary dropout, areas of nonperfusion, and neovascularization. OCTA has also proven useful in evaluating foveal avascular zone enlargement, which correlates with visual prognosis [54,55]. Recent advancements in wide-field OCTA have further expanded its diagnostic capabilities, enabling the comprehensive assessment of peripheral retinal ischemia and capillary non-perfusion, which are critical factors in determining the severity and progression of BRVO. Studies on wide-field OCTA have demonstrated its ability to provide an extended field of view, allowing clinicians to identify ischemic areas and peripheral neovascularization that may not be visible with conventional imaging methods. This capability informs more precise therapeutic interventions and enhances prognostic assessments, underscoring the importance of integrating wide-field OCTA into routine clinical evaluations of BRVO [54].

In the evaluation of BRVO, various diagnostic tools have been developed to assess structural and functional retinal changes, ischemic areas, and complications such as macular edema and neovascularization. Table 1 provides a comparative analysis of these diagnostic methods, highlighting their respective advantages and limitations. This overview aims to assist clinicians and researchers in selecting the most appropriate tools for accurate diagnosis and monitoring of BRVO [2,14,46,51,54,55,56,57,58,59,60,61,62].

Perimetry is a key functional diagnostic tool used to assess visual field defects in patients with BRVO. Specifically, microperimetry enables precise evaluation of retinal sensitivity at different points across the retina, providing localized functional information that correlates with structural changes observed through imaging techniques such as OCT. Microperimetry is particularly valuable in identifying early functional impairment, even in the absence of obvious changes in visual acuity, making it a critical tool for early detection and monitoring disease progression [46,51]. Overall, perimetry plays a vital role in evaluating retinal function, aiding in the management and tailored treatment strategies for BRVO patients.

In addition to imaging advancements, the study of biomarkers has gained prominence in predicting visual outcomes and tailoring treatment strategies for BRVO. Elevated levels of VEGF and inflammatory markers, such as interleukin-6, are closely associated with macular edema severity and treatment response [19]. Biomarker analysis offers valuable insights into disease mechanisms, enabling personalized treatment approaches tailored to individual patient needs. The integration of these advanced diagnostic tools into clinical workflows enhances the precision of BRVO diagnosis, facilitates early intervention, and supports tailored treatment plans, ultimately improving patient outcomes.

The differential diagnosis of BRVO includes several conditions that can present with similar clinical features. These conditions include ocular ischemic syndrome, proliferative diabetic retinopathy, hyperviscosity retinopathy, central retinal vein occlusion, papilledema, and acute hypertensive retinopathy. Accurate differentiation between these disorders is crucial for appropriate management and treatment strategies, as each condition requires distinct therapeutic approaches [50,63].

## 5. Therapy

The selection of treatment should be tailored to each individual, based on consultations among the patient, family, and physician. Addressing underlying medical conditions is essential to prevent occlusive events [64].

The treatment of BRVO should encompass three stages: identifying and managing risk factors, specifically treating vascular occlusion, and addressing BRVO complications, such as macular edema, retinal neovascularization, vitreous hemorrhage, and tractional retinal detachment [65].

The primary objective of all treatments is to resolve macular edema, the principal cause of impaired visual acuity and metamorphopsia, before the foveal photoreceptor layer is damaged [66,67]. The following are considered prognostic factors: patient age, baseline visual acuity and retinal thickness, early response to treatment, duration of macular edema, OCT characteristics, hyperreflective material, cytokine levels, central retinal sensitivity, microaneurysms in the perifoveal capillary network, retinal pigment epithelium integrity, retinal detachment, and intraretinal hemorrhage [68,69,70,71,72,73,74,75,76,77,78,79,80,81,82,83].

### 5.1. Medical Treatment

In BRVO, there is an increase in small platelet aggregates, which may contribute to the pathogenesis of this condition [10]. Although aspirin has been tested due to its anticoagulant properties, it has not improved vision. In fact, patients experienced poorer eyesight, more retinal bleeding, and greater visual loss [84]. Consequently, aspirin has not been recommended for ischemic patients [22].

Beraprost and ticlopidine inhibit the formation of small aggregates in patients with BRVO and may represent effective antiplatelet treatments [85]. It was found that ticlopidine significantly improved visual acuity in 69% of patients over a six-month period [86]. Additionally, it was reported that troxerutin significantly enhanced visual acuity in BRVO patients [10,87].

The use of low-molecular-weight heparins is deemed effective for treating BRVO, reinforcing the notion that BRVO is a venous thrombotic disorder. During treatment with low-molecular-weight heparins, no increased risk of vitreous hemorrhage was observed, and there was an improvement in visual acuity [10,88,89].

### 5.2. Isovolemic Hemodilution

BRVO is linked with hyperviscosity resulting from elevated hematocrit and plasma viscosity [34]. Blood viscosity is primarily influenced by hematocrit and plasma fibrinogen levels. In low circulation conditions, such as in an occluded vein, the impact of viscosity becomes more pronounced due to increased erythrocyte aggregation. Furthermore, hypoxia induced by occlusion further exacerbates blood viscosity, as acidosis promotes erythrocyte aggregation and diminishes erythrocyte elasticity [90,91]. Hemodilution, achieved by reducing hematocrit to a target value of 35%, has been shown to be beneficial in the early phase, specifically within the first two weeks following the onset of RVO. This approach can improve visual acuity by 43% even after RVO has been established, with favorable long-term outcomes [64]. However, the systemic complications associated with this method render it less preferable compared to other available therapies [10].

### 5.3. Laser Therapy

Laser photocoagulation is regarded as the standard treatment for neovascular complications associated with RVO, including retinal and optic disc neovascularization, as well as iris neovascularization. The Branch Vein Occlusion Study demonstrated that focal laser photocoagulation significantly improves vision in patients with macular edema secondary to branch retinal vein occlusion. This study established grid laser therapy as the primary treatment for BRVO-related macular edema for subsequent decades. Notably, 65% of participants in the grid laser group experienced a gain of ten or more lines of letters 36 months after treatment [27,47].

Conventional laser therapy can result in complications such as enlarged retinal scars, subretinal fibrosis, choroidal neovascularization, and reduced macular sensitivity. Its effectiveness is consistently surpassed by anti-VEGF therapy and corticosteroids [92,93,94].

Subthreshold laser and micropulse laser therapy are modern retinal laser therapies used in the management of BRVO, particularly for addressing macular edema. Subthreshold laser therapy is performed at lower intensity than traditional laser treatments, providing therapeutic benefits without causing visible burns or scarring to the retina. This technique is effective in reducing macular edema in BRVO, promoting improved visual outcomes while preserving the structural integrity of the retina. Studies have shown that subthreshold laser therapy can be a safe alternative to conventional laser photocoagulation, with reduced side effects and a lower risk of complications such as vision loss and retinal damage. Similarly, micropulse laser therapy delivers laser energy in short pulses, allowing for intermittent cooling of the retina. This method minimizes the thermal damage associated with traditional continuous-wave lasers and is particularly beneficial for treating macular edema in BRVO. Both subthreshold and micropulse lasers have been shown to effectively reduce edema and improve visual acuity, making them valuable tools in the management of BRVO, offering safer and more precise alternatives to conventional laser treatments [27,92]. Their adoption into clinical practice has the potential to enhance patient outcomes, particularly for those unresponsive to pharmacological therapies or ineligible for frequent intravitreal injections.

A comparative study revealed that intravitreal anti-VEGF therapy achieved significantly better outcomes than focal laser photocoagulation [51]. Consequently, anti-VEGF therapy has become the preferred first-line treatment for macular edema [10]. However, laser therapy remains relevant for specific cases, such as managing peripheral neovascularization or as an adjunct for patients who are refractory to anti-VEGF treatment [92].

### 5.4. Anti-VEGF Therapy

VEGF inhibitor therapy is an effective approach for managing macular edema and treating neovascularization resulting from RVO. Various anti-VEGF agents have been studied and are utilized according to different treatment regimens. Some specialists advocate for monthly injections, while others prefer a treat-and-extend or as-needed (pro re nata) regimen [95,96,97,98,99,100]. Currently available anti-VEGF therapies are compiled in Table 2 [101,102,103,104,105,106,107,108,109,110,111].

Among anti-VEGF therapies, brolucizumab has been noted for its potential benefits, particularly in reducing injection frequency due to its extended dosing intervals. Case reports suggest that brolucizumab effectively reduces macular edema and improves visual acuity in patients with BRVO. Brolucizumab’s safety profile has raised concerns, as it has been associated with serious adverse events, including intraocular inflammation, retinal vasculitis, and retinal artery occlusion. However, its use remains off-label in this context and is supported by limited evidence [109].

Intravitreal anti-VEGF injections, while highly effective for treating retinal conditions, can lead to various complications. Table 3 outlines these complications, their management, and relevant references [112,113,114].

### 5.5. Intravitreal Corticosteroid Therapy

Intravitreal corticosteroid therapy effectively inhibits various immunomodulators and VEGF [27]. This treatment modality is particularly advantageous in cases where the financial burden of anti-VEGF therapies is prohibitive for patients. Among the corticosteroids, triamcinolone acetonide is frequently utilized and has been reported to yield comparable or superior short-term outcomes, particularly in non-ischemic BRVO, relative to anti-VEGF treatments [10].

Current evidence supports the use of corticosteroids as a secondary therapeutic option for patients with RVO. They are recommended as an alternative for individuals who have not shown sufficient response to anti-VEGF injections, typically after a series of three to six injections. Corticosteroids may also be considered as a first-line therapy for patients with recent significant cardiovascular events or for those who are unable to adhere to the frequent monthly visits required for anti-VEGF therapy and monitoring within the initial six months. Nevertheless, IOP should be regularly monitored following corticosteroid injections [51].

Intravitreal corticosteroids, notably the intravitreal implant containing dexamethasone, have been associated with a prompt improvement in visual acuity. However, this treatment is accompanied by notable side effects, including increased intraocular pressure and the development of cataracts [115,116]. The GENEVA study, which evaluated intravitreal dexamethasone implants for BRVO, reported a mean gain of 15 letters in visual acuity at 180 days. The study also observed that 29% of eyes treated with repeated dexamethasone implants experienced cataract progression, in contrast to 6% of eyes that received only a single implant [27].

The use of fluocinolone acetonide (FAc) intravitreal implants has been explored as an off-label treatment for macular edema secondary to RVO, including BRVO and CRVO. FAc implants deliver sustained-release corticosteroid therapy, reducing inflammation and stabilizing the blood–retinal barrier over extended periods. In a systematic review, the 0.19 mg FAc implant demonstrated efficacy in improving visual acuity and reducing macular thickness in patients with refractory or chronic macular edema associated with RVO. The review highlighted that these implants offer a viable option for patients unresponsive to first-line therapies, such as anti-VEGF agents [117].

Corticosteroid therapy is a pivotal treatment option for managing macular edema in BRVO, particularly in patients who are unresponsive to anti-VEGF agents or have contraindications to their use. Despite their efficacy, corticosteroids are associated with several complications that require vigilant management. Elevated intraocular pressure (IOP) is a common adverse effect, potentially leading to steroid-induced glaucoma if left untreated. Prolonged corticosteroid use can also result in cataract formation, particularly posterior subcapsular cataracts, necessitating surgical intervention in advanced cases. In rare instances, intraocular inflammation, such as sterile endophthalmitis, and secondary infections may occur, especially in immunocompromised individuals. These risks highlight the importance of individualized treatment plans, careful patient selection, and routine monitoring to optimize therapeutic outcomes while minimizing adverse effects [112,113,114,115,116].

### 5.6. Surgical Intervention

Given the understanding of the mechanisms underlying vein occlusion, arteriovenous sheathotomy presents a theoretically sound approach for treating BRVO. In 1988, Osterloh and Charles introduced a surgical technique involving the dissection of the common adventitial sheath at the location of the arteriovenous occlusion, aiming to alleviate the pressure at the arteriovenous crossing. Arteriovenous sheathotomy not only reduces the pressure at the crossing point but also diminishes interleukin-6 (IL-6) expression. Nonetheless, the efficacy of this procedure remains a subject of debate, with conflicting reports regarding its overall effectiveness [118,119].

Clinical studies have demonstrated that vitrectomy can effectively manage complications of RVO, such as vitreous hemorrhage and retinal detachment. Research indicates that vitrectomy can lead to substantial improvements in visual acuity and resolution of hemorrhage, although outcomes vary based on the extent of retinal damage and preoperative conditions [120].

### 5.7. Combined Therapy

There is limited evidence supporting the notion that combination therapy offers additional benefits over single-agent treatments. Nonetheless, some researchers advocate that combining two or more therapeutic agents may enhance outcomes by reducing the frequency and dosage of individual treatments. Repeated intravitreal injections of anti-VEGF agents can lead to complications such as ocular pain, ischemic retinopathy, and endophthalmitis, in addition to the high costs associated with drugs like ranibizumab. In this context, it has been observed that combining bevacizumab with macular grid photocoagulation can substantially improve visual acuity, diminish macular edema, and better prevent the recurrence of CME compared to bevacizumab alone [10]. Recent research has assessed the efficacy of dexamethasone implant, a corticosteroid implant, compared to a combination therapy involving anti-VEGF agents and corticosteroids in treatment-naive cases of branch retinal vein occlusion with macular edema. The study underscores the advantages of combining anti-VEGF therapy with cortisone (Vitreal S), which provides a more cost-effective alternative while achieving results comparable to those of dexamethasone implant therapy [121].

Furthermore, the topical application of bromfenac, an NSAID, in conjunction with intravitreal bevacizumab treatment for macular edema secondary to BRVO, has been reported to decrease the required number of injections [10].

Other combination therapies that have been explored include the use of laser treatment in conjunction with corticosteroids, corticosteroids combined with anti-VEGF agents, laser therapy paired with anti-VEGF agents, and corticosteroids combined with arteriovenous sheathotomy or pars plana vitrectomy. While many of these studies have reported favorable outcomes, it remains challenging to make direct comparisons between them to determine the most effective combination therapy [10].

## 6. Actualities

### 6.1. Gene Therapy

Gene therapy represents a promising frontier in the treatment of BRVO by targeting the underlying mechanisms of the disease at a molecular level, such as VEGF overexpression. Gene therapy techniques aim to modulate the expression of VEGF, a critical player in BRVO pathogenesis. Strategies include using gene transfer to deliver VEGF inhibitors directly to the retina [122]. Techniques such as CRISPR/Cas9 are being investigated to correct genetic mutations that contribute to retinal diseases, potentially offering new avenues for treating BRVO [123].

Several clinical trials are underway to evaluate the safety and efficacy of gene therapy. Early results suggest that gene therapy could provide long-lasting effects by reducing the need for repeated injections [124]. Despite its potential, gene therapy faces challenges including delivery methods, potential immune responses, and the need for long-term safety data [125].

### 6.2. Peptide-Based Agents

Peptide-based agents represent an innovative approach in the treatment of BRVO, offering targeted therapeutic strategies aimed at modulating specific molecular pathways involved in the disease. Peptide-based agents are designed to target specific proteins or signaling pathways involved in retinal inflammation and angiogenesis. By interfering with these pathways, these agents aim to reduce retinal edema and improve visual outcomes [126]. Some peptide-based agents act as anti-inflammatory agents, targeting cytokines and other inflammatory mediators that contribute to BRVO pathology [127].

Peptides can also inhibit angiogenic factors that promote abnormal blood vessel growth, which is a key feature in BRVO. By blocking these factors, peptide-based agents may prevent the progression of the disease and improve visual acuity [128].

Initial studies on peptide-based agents have shown promising results in reducing macular edema and improving visual acuity. Ongoing clinical trials are evaluating their safety, efficacy, and optimal dosing regimens [129]. The development of peptide-based therapies faces challenges such as efficient delivery, potential immunogenicity, and the need for extensive clinical validation [130].

### 6.3. Small-Molecule Inhibitors

Small-molecule inhibitors represent a promising approach in the treatment of BRVO by targeting various intracellular pathways and molecular mechanisms involved in the disease. These agents are designed to provide more precise and often less invasive treatment options compared to traditional therapies. Small-molecule inhibitors are designed to target intracellular signaling pathways activated by VEGF, which plays a crucial role in retinal edema and neovascularization in BRVO. These inhibitors work by blocking the downstream effects of VEGF signaling [131]. Certain small molecules specifically target inflammatory pathways, thereby reducing the production of cytokines and other inflammatory mediators that contribute to retinal damage and edema [132]. Furthermore, by inhibiting pathways involved in fibrosis, these agents can help prevent scar tissue formation and preserve retinal function [133]. Moreover, small molecules can modulate vascular permeability in the retina, reducing leakage and edema associated with BRVO [134].

Some researchers underscore the significance of metabolomic role in the context of retinal vascular diseases, suggesting that it could enhance our understanding of disease mechanisms and lead to better management strategies for conditions like RVO [135]. However, the development of these small-molecule inhibitors presents several challenges. Key issues include ensuring effective delivery to the retina, minimizing off-target effects, and establishing long-term safety and efficacy through comprehensive clinical trials [136].

## 7. Discussion

The central challenge associated with retinal vein occlusion is vision loss, particularly critical in elderly populations concerned with restoring visual acuity. Numerous studies have demonstrated that the type of occlusion significantly influences the prognosis of visual acuity, alongside the patient’s age. Additionally, several researchers emphasize the importance of other prognostic factors, such as central macular thickness, hyper-reflective foci, foveal intraretinal hemorrhage, disruption of the ellipsoid zone, integrity of the inner retinal layers, and vitreomacular adhesion [53,70,75,76].

While laser photocoagulation was historically the standard treatment, its effectiveness in improving visual outcomes is limited compared to newer therapies. Anti-VEGF agents and intravitreal corticosteroids have emerged as the cornerstone treatments for macular edema secondary to BRVO. However, macular edema recurrence and resistance to therapy remain unresolved challenges. A combination of anti-VEGF agents and corticosteroids or adjunctive therapies, such as subthreshold or micropulse lasers, may offer alternative approaches for patients who do not respond to monotherapy.

Complications from intraocular injections can arise due to the host’s reaction, the drug’s effects, or the injection technique. Research suggests that the most common complications are typically minor and often resolve spontaneously without the need for special treatment. Potential complications of intravitreal injections encompass corneal abrasions, surface irritation, subconjunctival hemorrhages, cataract formation, vitreous hemorrhage, retinal tears and detachment, elevation of intraocular pressure, intraocular inflammation, and in severe cases, endophthalmitis [137]. All in all, intraocular injections are a safe therapeutic option for managing branch retinal vein occlusion, with the majority of associated complications being minor and self-limiting.

Emerging therapies, such as gene therapy, peptide-based agents, and small mole-cule inhibitors, represent promising advancements. Gene therapy aims to address BRVO at a molecular level, potentially offering sustained benefits with fewer interventions. Peptide-based agents target angiogenic and inflammatory pathways, while small-molecule inhibitors focus on intracellular signaling to reduce vascular permeability and inflammation. Although early trials are encouraging, these approaches face challenges, including delivery methods and long-term safety validation [122,123,124,125,126,127,128,129,130,131,132,133,134,135,136].

In conclusion, while substantial progress has been made in the treatment of BRVO, several challenges remain. These include addressing therapeutic resistance, reducing recurrence rates, and enhancing personalized treatment strategies. Future research should focus on integrating innovative therapies, such as gene therapy and small-molecule inhibitors, into clinical practice, aiming to improve long-term outcomes and the quality of life for patients with BRVO.

## Figures and Tables

**Table 1:** Advantages and limitations of diagnostic methods for BRVO.

Method	Advantages	Limitations
**Fundus photography**	- Provides clear structural visualization of the retina, aiding in the assessment of retinal hemorrhages, neovascularization, and optic disc changes - Widely available and cost-effective for initial screenings	- Lacks depth specificity and functional insights, requiring complementary imaging for comprehensive evaluation - Does not provide dynamic or vascular flow data
**Fluorescein angiography**	- Gold standard for identifying ischemia, vascular leakage, and neovascularization - Offers detailed visualization of microvascular changes and capillary non-perfusion	- Invasive procedure with potential side effects such as nausea and allergic reactions; unsuitable for some patients - Limited depth specificity and significant time and resources required
**Optical coherence tomography**	- Non-invasive imaging with high resolution to detect macular edema, retinal thickness changes, and subretinal fluid - Quantifies central retinal thickness, guiding therapeutic decisions and monitoring responses to treatment	- Limited capability for visualizing deeper retinal and choroidal vasculature
**OCT angiography**	- Non-invasive imaging of superficial and deep capillary plexuses; detects capillary dropout and non-perfusion areas in ischemic BRVO - Enables differentiation between ischemic and non-ischemic BRVO, and evaluates foveal avascular zone enlargement	- Narrow field of view; cannot detect leakage areas or fully assess vascular permeability - Limited capability for choroidal and choriocapillaris visualization
**Scanning laser ophthalmoscopy**	- Provides high-resolution real-time imaging, offering detailed structural views of smaller capillaries and retinal layers	- Limited depth resolution; difficulty distinguishing between vascular beds
**Multimodal OCT and photoacoustic microscopy**	- Combines structural and functional imaging with enhanced spatial resolution, enabling subtle evaluations of blood flow and vessel integrity	- Requires costly equipment, complex protocols, and contrast agents for optimal sensitivity and specificity

**Table 2:** Anti-VEGFs used for the treatment of BRVO.

Anti-VEGF Agent	Structure and Mechanism of Action	Treatment Interval	Efficacy	Associated Complications
**Bevacizumab** **(Avastin)**	Full-length monoclonal antibody; targets VEGF-A	Monthly or as-needed (PRN)	Effective for reducing macular edema and improving visual acuity; used off-label for BRVO	Risk of systemic complications, mild intraocular pressure, or inflammation
**Ranibizumab** **(Lucentis)**	Monoclonal antibody fragment; targets VEGF-A	Monthly or treat-and-extend	Significant improvement in visual acuity and reduction in macular edema; FDA-approved for BRVO	Mild intraocular pressure or inflammation
**Aflibercept** **(Eylea)**	Recombinant fusion protein of VEGF; targets VEGF-A and PlGF	Treat-and-extend approach after initial loading doses	Effective in reducing macular edema and improving visual acuity; allows for longer intervals between injections; FDA-approved for BRVO	Mild intraocular pressure or inflammation
**Brolucizumab** **(Beovu)**	A single-chain variable fragment; targets VEGF-A	Every 6–8 weeks after initial loading doses	Newer agent with potential for fewer required injections; promising results in recent studies; used off-label for BRVO	Intraocular inflammation, retinal vasculitis, retinal artery occlusion
**Faricimab** **(Vabysmo)**	Bispecific monoclonal antibody; targets VEGF-A and Ang-2	Every 8 weeks or up to 16 weeks after initial doses	Demonstrated efficacy in reducing macular edema with extended dosing intervals; FDA-approved for BRVO	Mild intraocular inflammation, systemic thromboembolic events

**Table 3:** Complications of the intravitreal anti-VEGF injections.

Complication	Complication Rates	Management
Increased IOP	Transient IOP spikes are common (10–50%), but long-term elevation occurs in about 2–5%	Regular monitoring and management with antiglaucoma medications if needed
Subconjunctival hemorrhage	10–40%	Typically resolves on its own
Corneal damage	0.01–0.1%	Typically resolved with minimal intervention
Cataracts	More commonly associated with intravitreal corticosteroid injections than anti-VEGF agents (**15–40%**)	Surgical intervention
Vitreous hemorrhage	0.02–0.1%, more likely in patients with pre-existing conditions such as retinal neovascularization	May require surgery
Infection (endophthalmitis)	0.01–0.09%	Sterilization, prophylactic antibiotics
Ocular ischemia	<0.01%	Monitoring and treatment
Systemic effects	<1%, more likely in patients with pre-existing cardiovascular or cerebrovascular disease	Management of systemic reactions as they arise
